# Development of a pH‐sensing indicator for shrimp freshness monitoring: Curcumin and anthocyanin‐loaded gelatin films

**DOI:** 10.1002/fsn3.3375

**Published:** 2023-04-17

**Authors:** Fatemeh S. Mohseni‐Shahri, Farid Moeinpour

**Affiliations:** ^1^ Department of Chemistry, Bandar Abbas Branch Islamic Azad University Bandar Abbas Iran

**Keywords:** food spoilage sensor, intelligent packaging, mixed natural dyes, pH indicators

## Abstract

An intelligent pH‐sensing indicator containing Roselle (*Hibiseus sabdariffa* L.) (RS) anthocyanin and curcumin (CR) was developed and characterized as on‐package indicator tags to check the freshness of shrimp during the storage at 4°C. FE‐SEM and FT‐IR analysis showed that RS and CR were successfully immobilized in the gelatin–glycerol film‐forming substrate. The addition of various natural dyes increased the thickness and antioxidant action of the colorimetric film. To assess the response to changes in the pH, the colorimetric film was immersed in different buffers. Based on volatile amines secreted by shrimp, a test application of a colorimetric film containing natural dyes at a ratio of CR:RS = 1:4 (v/v) was conducted in shrimp at 4°C. The total volatile basic nitrogen (TVB‐N) and the pH of shrimp were monitored during refrigerated storage for 10 days, and the color changes of the indicator were recorded simultaneously. The results indicated that the designed colorimetric film could produce various colors, which are thought to be indicative of the freshness and spoilage of packaged shrimp. Therefore, the target film can be utilized as a promising smart packaging material for monitoring the freshness of shrimp and aquatic products in real time.

## Graphical abstract

Results indicated that a mixture of curcumin and anthocyanin could be a good indicator material for intelligent film to nondestructively monitor the freshness of shrimp.

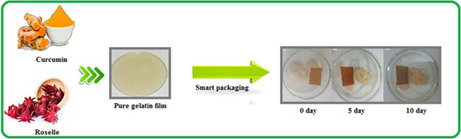

## 1 INTRODUCTION

Human society is growing in such a way that the demand for food is increasing every year, hence, food production is also increasing rapidly (Mercier et al., 2017). On the other hand, many foods, due to their high sensitivity to environmental factors, must be placed in their own packaging immediately after production, and non‐sensitive foods also need more ordinary packaging for distribution. The most important purpose of ordinary packaging is to separate the food from the environment and prevent it from meeting external contaminants or spoilers. However, these characteristics do not meet the needs of the food industry in today's society (Müller & Schmid, 2019). Most advances in modern packaging products are designed in a way that leads to lower costs, better and more access, and reduced food quality. Today, to improve food packaging, various solutions are offered that have three important aspects of antimicrobial properties, the most suitable materials for packaging production and production techniques (Yildirim et al., 2018). One of the new types of packaging is smart packaging. The most prominent feature of this type of packaging is the sensitivity to environmental changes during the storage period of food so that the packaging set automatically shows a suitable feature to the expected adverse changes (Nemecek & Jungbluth, 2016). In fact, intelligent packaging is a system that is able to perform intelligent functions (such as detect, sense, record, tracking, and communication) to facilitate decisions to increase expiration date, enhance health, ameliorate quality, prepare data and inform about probable difficulties (Chowdhury & Morey, 2019). In most cases, these packages have indicators that are placed on the surface of the package and can play an informing and warning role for the manufacturer or consumer. These smart packages work in such a way that the markers in them are sensitive to some things and react at the proper time. These include changes in temperature and when food storage conditions are not appropriate (Taoukis & Labuza, 1989). With this function, smart packaging indicators can inform the producer, seller, and consumer about the freshness or spoilage status of foods and also the resulting food poisonings. It should be noted that in some cases, poisoning can even lead to their death (Biji et al., 2015). Nevertheless, nature has always been a good example for human beings. By modeling natural phenomena, researchers can extract the best mechanisms and engineering from them and use them artificially in various applications (Boo et al., 2012). As is well known, spoilage of meat foods or fishery products always produces relatively great TVB‐N (total volatile basic nitrogen viz., dimethylammonium, ammonia, and trimethylamine), which can be easily shown by colorimetric film with the ability to detect pH by visible color changes. Lately, many indicators have been developed to monitor the freshness of the common seafood products, such as fish (Moradi et al., 2019) and shrimp (Kang et al., 2020; Liu et al., 2019; Ma et al., 2017) or meat foods, such as bacon (Choi et al., 2017; Zhang, Zou, et al., 2019).

Anthocyanins from flowers, vegetables, and fruits are broadly utilized for the development of colorimetric indicators owing to the their safety and pH‐sensing properties, like those of blueberries, (Luchese et al., 2017) red cabbage, (Liang et al., 2019) black soybean seed coat, (Wang, Yong, et al., 2019) and purple sweet potato (Yong et al., 2019).

Kan et al. (2022) used 14 plants as anthocyanin sources and immobilized their extracts in starch/polyvinyl alcohol matrices and prepared freshness monitoring labels. The results showed that the microstructural, optical, antioxidant, and pH‐sensitive properties of anthocyanins‐rich labels are strongly affected by the anthocyanin source (Kan et al., 2022; Sinela et al., 2017).

*Hibiseus sabdariffa* L. (Roselle) (RS) is an herbal plant, grown mostly in tropic and subtropic regions of both hemispheres (Degenhardt et al., 2000). Its calyx contains large amounts of anthocyanins up to 1.5 g/100 g on dry weight (Peralta et al., 2019). The biological activities of RS anthocyanin, such as antioxidant activity and antihypertensive effect have been studied and limited studies have been conducted on the use of its extract alone to fabricate a pH indicator. Zhai et al. (2017) observed that the cross‐sectional morphology of the starch/PVA label with extract became more and more uniform when the content of anthocyanins‐rich RS extract increased. However, the potential use of RS for the development of mixture colorimetric indicators has not been explored.

Many studies have concentrated on CR (curcumin), which is elicited from the turmeric. It is a biologically active member of curcuminoids and is broadly utilized as a colorant and spice (Kharat et al., 2017; Wang, Xue, & Zhang, 2019). CR is a lipid‐soluble phytochemical with pH‐dependent solubility (Kuswandi et al., 2012). Its extract has been used in the fabrication of various pH sensitive indicators (Liu et al., 2018; Ma et al., 2017; Mohseni‐Shahri et al., 2023).

In comparison with a singular indicator, a mixture indicator can extend the domain of color chang (Rukchon et al., 2014). For instance, a mixture‐pH dye indicator on the basis of a synthetic pH‐sensitive dye (e.g., a mixture of methyl red and bromothymol blue) has been developed to provide a more correct indicator system for monitoring nondestructive and real‐time freshness of skinned chicken breasts (Qin et al., 2020). Qin et al. conducted studies on smart packaging by adding anthocyanin or betacyanin or anthocyanin/betacyanin mixture to starch/polyvinyl alcohol films and compared the physical and functional properties of the films. Results showed that among different films, the film containing anthocyanins/betacyanins mixture in the weight ratio of 1:3 presented obvious color changes when the film was utilized to monitor the freshness of pork (Sindi & Marshall, 2014).

Therefore, mixtures of CR and RS were chosen for the development of a new colorimetric pH‐sensor film in this research. The color alteration of mixed natural dyes against changes in pH was studied and the impact of various dye formulations on ammonia was investigated. Eventually, the possible utilization of colorimetric films has been realized in shrimp freshness monitoring.

## 2 EXPERIMENTAL

### 2.1 Materials

Sun‐dried *Hibiscus sabdariffa* (RS) flowers were obtained from the native marketplace in Bandar Abbas, Iran. Curcumin, glycerol, ammonia solution, acetic acid, and ethanol were purchased from Sigma‐Aldrich Co. Gelatin powder was obtained from Surechem Products (SCP) Co. All chemicals were of analytical grade and deionized water was used to prepare the solutions.

### 2.2 Extraction of anthocyanins from *Hibiscus sabdariffa* (RS) flowers

*Hibiscus sabdariffa* (RS) flowers were ground into a fine powder using a household blender. Dried RS powder (1.0 g) was elicited with solvent (100 mL of methanol, water, and ethyl acetate). Each extraction mixture was heated at 25°C, 50°C, or at the boiling point of the solvent for 3, 5, or 10 min. The ultimate extract was gained by filtration through a Whatman No. 1 filter paper (Wrolstad, 1993).

The exact anthocyanins content of the extract was calculated according to the method described by Wrolstad (1993) and Yang et al. (2015) and was 243.54 mg/L.

### 2.3 Spectral characteristics of natural dye solutions

Curcumin (CR) dye was prepared as follows: 1 g/L curcumin in acidified ethanol (a mixture of 1.0 mol/L acetic acid and at a ratio of 3:17).

A volume of 50 μL of 1 g/L natural dye solution (CR, RS, and CR and RS mixed solutions in 1:1 (v/v), 1:4 (v/v), or 4:1 (v/v)) was added to 5 mL of pH buffer. The spectra of the natural dye solutions at various pHs (2–12) were then measured using a spectrophotometer (Specol 1500‐ Analytical Jena AG) in the range of 400–800 nm.

### 2.4 Preparation of the colorimetric films

The film formative solution was prepared as follows: two aqueous solutions were initially prepared by magnetic stirring, one containing 10% (w/v) gelatin at 100°C and a solution containing 2.5% (w/v) glycerol plus 5% (v/v) mixed CR and RS solutions in a ratio of 1:4 (v/v) at room temperature. Then equal volumes of both solutions were mixed by shaking for an additional 30 min at room temperature. Eventually, 10 mL of each film formative solution was poured onto a Petri dish (9.0 cm) and dried in an oven at 60°C for 3 h.

### 2.5 Characteristics of the colorimetric films

#### 2.5.1 FT‐IR (Fourier transform infrared) spectroscopy

FT‐IR spectra of the films were acquired with a FT‐IR spectrometer (Nicolet IS10, Thermo Nicolet Corporation, USA) in the range of 400–4000 cm^−1^ at a resolution of 4 cm^−1^ and the scan number was 16.

#### 2.5.2 Water content, swelling index, water solubility, and thickness of the colorimetric films

Moisture content was determined by heating to constant mass at 105°C in a hot air oven and calculated as follows (Gontard et al., 1992):

(1) $$\text{Moisture content}\;\left(\%\right)=\left(m–M\right)/m\times 100$$

where *m* is the weight of the film before drying and *M* is the constant weight after drying. Water solubility was tested as follows: 3 cm × 3 cm of the film was cut, weighted, and soaked in 50 mL of water at 25°C for 24 h. Then, the water was eliminated, and the remained film was dried at 105°C to constant weight. The dry weight of the primary film was computed as stated in formula (1). Water solubility was computed by the following formula (Tirtashi et al., 2019):

(2) $$\text{Water solubility}\;\left(\%\right)=M–W/M\times 100$$

where *M* is the dry weight of the initial film and *W* is the dry weight of the remaining film. Immersion method in an aqueous medium was used to evaluate the swelling of colorimetric film. Pieces (5.5 × 15 mm) were prepared from the film and its initial mass was determined in terms of grams with a precision of 0.0001 g. The samples were put in Petri dishes comprising distilled water (30 mL) and kept at 25°C. After 20 min, the samples were removed from the petri dish at different times and their excess water was gently filtered with filter paper and the final weight of the films was determined. The swelling index percent was computed using the Equation (3) (Zhai et al., 2017).

(3) $$\text{Swell index}\;\left(\%\right)=\left(\left(W_{2}–W_{1}\right)/W_{1}\right)\times 100$$

in which *W*_1_ indicates the weight of the dry sample and *W*_2_ represents the weight of the swollen sample.

The thickness of the packaging films was determined at several different points and randomly with a digital micrometer with an accuracy of 1.00 μm and then their average was calculated.

#### 2.5.3 Mechanical properties of the film

The mechanical properties of the films, including maximum tensile strength (TS), elongation to breaking point (EB), and elastic modulus (EM), were performed based on the approved standard ASTM D 882–88 and the Universal testing machine. Before performing the test, the samples were conditioned for 24 h in terms of humidity and finally, the mechanical properties of the films were calculated from the following relationships:

(4) $$\text{Tensile strength}\;\left(\mathrm{MPa}\right)=\mathrm{Max}\;\text{load}/\text{Cross}-\text{sectional of area sample}$$

(5) $$\text{Elongation}\;\mathrm{at}\;\text{break}\;\left(\%\right)=\text{Elongation}\;\mathrm{at}\;\text{break point}/\text{original length}$$

#### 2.5.4 Investigating the morphological and structural characteristics of films

The morphological characterization of the surface of pure and anthocyanin‐incorporated gelatin films was studied by a TESCAN BRNO‐Mira3LMU FE‐SEM electron microscope (TESCAN, Czech Republic).

#### 2.5.5 Color response to volatile NH_3_

Color responses of the colorimetric film to volatile NH_3_ were conducted using a previously reported procedure with minor corrections (Chen et al., 2019; Peralta et al., 2019) 2 cm × 2 cm of the film was cut and hung up in a reagent bottle (500 mL) at 1 cm above the 80 mL of 0.008 mol/L ammonia solution at ambient temperature. The pictures and colorimetric information of the films were registered every 2 min for 20 min as stated in the procedure explained in the literature (Ohta, 1977). Image analysis was performed by Photoshop software. The total color variation (Δ*E*) was computed by the formula (6) (Goulas & Kontominas, 2005):

(6) $$∆E=\sqrt{{\left(L^{*}-L_{0}^{*}\right)}^{2}+{\left(a^{*}-a_{0}^{*}\right)}^{2}+{\left(b^{*}-b_{0}^{*}\right)}^{2}}$$

where $L_{0}^{*}$, $a_{0}^{*}$ and $b_{0}^{*}$ are numeric values for luminance (white to black), red to green color range, and yellow‐to‐blue color range, respectively obtained at 0 min, and L*, a*, and b* are the values at the time of sampling.

The related color change is computed as follows (Chen et al., 2019):

(7) $$S\left(\%\right)=ǀR–R_{0}ǀ+ǀG–G_{0}ǀ+ǀB–B_{0}ǀ/\left(R+G+B\right)$$

where *R*_0_, *G*_0_, and *B*_0_ are the values registered at 0 min, and *R*, *G*, and *B* were the values at the time of sampling.

### 2.6 Spoilage monitoring of shrimp

Shrimp samples (10 ± 1 g) were placed in a sterilized Petri dish (9.0 cm). After being sealed with plastic wraps, they were kept for 10 days at 4°C. Indicator film was adhered inside the cap of the petri dish which had no contact with the packaged shrimp. The Δ*E* values of the indicator were calculated according to the method described above.

### 2.7 pH

To determine the pH value, 10 g sample of the shrimp meat was homogenized (at 1000 rpm for 1 min) in 90 mL of distilled water and the resulting mixture was filtered. Then, the pH of the samples was measured using a digital pH meter (EDT DIRECTION pH meter model GP353) after 5 min incubation at ambient temperature (Lotfi et al., 2018).

### 2.8 Total volatile base‐nitrogen (TVB‐N)

TVB‐N was measured based on a steam distillation method (Ma et al., 2017). Homogenized shrimp was distilled after the addition of MgO. The distillate was collected in a flask containing an aqueous solution of boric acid and methyl red as an indicator, and then titrated with sulfuric acid solution. TVB‐N contents were described as mg/100 g and estimated as follows:

(8) $$\mathrm{TVB}-N\;\text{contents}\;\left(\mathrm{mg}/100\;g\right)=V_{1}\;\left(\mathrm{mL}\right)\times 14$$

where *V*_1_ is the volume of sulfuric acid used in the titration.

### 2.9 Statistical data processing

All measurements were conducted three times. The statistical data processing was performed using IBM SPSS Statistics 20.0. An ANOVA (analysis of variance) using Tukey's multiple range test was performed for mean comparison at *p* < .05.

## 3 RESULTS AND DISCUSSION

### 3.1 Visible spectra of natural dyes at various pHs

**FIGURE 1:**
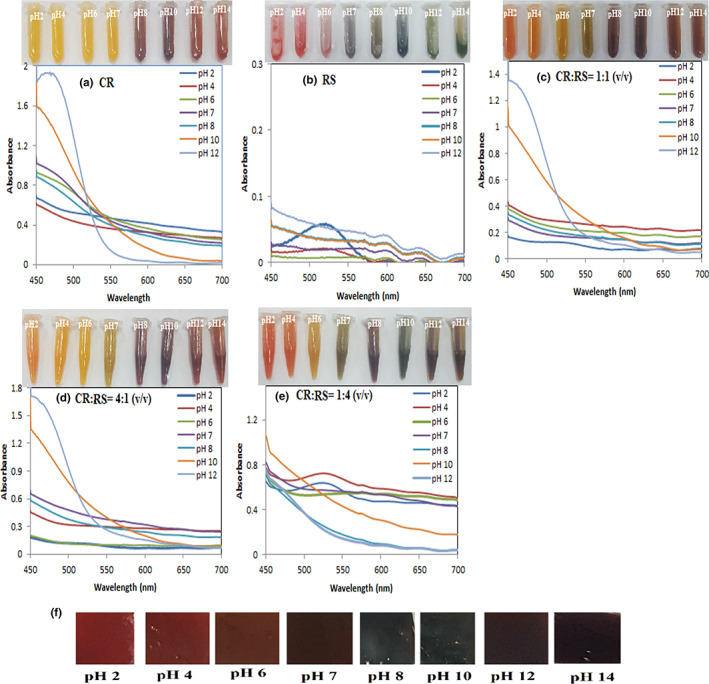
Color and visible spectra of (a) CR, (b) RS, and (c–e) mixture of CR and RS at different pHs, (f) Color changes of the fabricated colorimetric film (mixing CR and RS in a ratio of 1:4 (v/v)) at different pHs.

**FIGURE 2:**
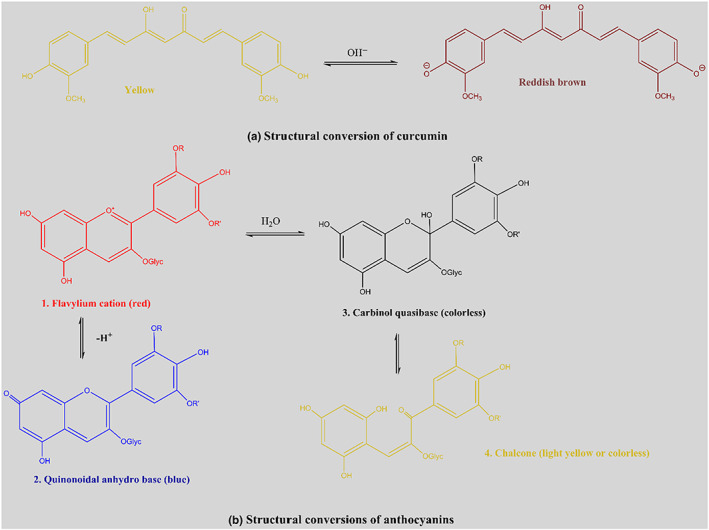
Structural transformations of (a) curcumin and (b) anthocyanins in various buffer solutions.

To accredit the natural dyes solutions to use as pH indicator dyes, color variations of them were tested in different pH buffer solutions. As can be seen in Figure 1a, the color of CR was yellow when pH was <8, brown at pH 8–10, and light brown at pH 12–14. Figure 2a indicates a schematic of the reversible structural changes of CR at various pH values (Esatbeyoglu et al., 2012). In acidic and neutral solutions, the keto form of CR dominates, while at pH values higher than 8, the enol form preponderates (Grajeda‐Iglesias et al., 2016). The absorption spectra of CR versus pH changes showed different side views.

Bathochromic shifts in maximum absorption peaks were mainly caused by the structural transformation of curcumin at different pH values. Anthocyanin is a polyphenolic that has an acid–base indicator property (Tirtashi et al., 2019). As could be observed in Figure 1b, the color of RS was pinkish purple at pH 2–4, violet at pH 6, blue at pH 7, and green at pH 8–14. The color change was dependent on the variation of the RS structure (Figure 2b). By increasing the pH from 2 to 12, the utmost absorption wavelength of RS displaced from 520 nm to 595 nm. These shifts might be a result of the reversible structural alterations of RS in acidic to an alkaline aqueous solution (Chen et al., 2020). As could be observed in Figure 1a,b CR indicated less color changes in comparison to RS.

The pH‐sensitive dye mixture provided by mixing CR and RS in 1:1 (v/v) ratio is indicated in Figure 1c. At a pH below 7, the color of the mixed dye is pale brown, and the maximum absorption peak shifted from 452 nm to 449 nm.

Figure 1d shows the color change of pH‐sensitive mixture dye provided by mixing CR and RS in a 4:1 (v/v) ratio at various pHs. The color change and maximum absorption peak shift of the mixture were approximately like the color change of CR lonely. Mixed pH‐sensitive dye prepared by mixing CR and RS in a 1:4 (v/v) ratio is indicated in Figure 1e, in which color changes in various pHs are completely different and recognizable by the naked eye. By raising the pH, the maximum absorption wavelength of the mixed dye was transferred from 530 nm to 607 nm.

Table 1 presents the color variables (L*, a*, b*, and △*E*) of CR, RS, and the mixing CR and RS in different ratios solutions in pH 2–14 solutions. The results indicated that a mixture indicator can extend the range of color change compared with a single indicator. Due to the expanded range of color change and color stability over time, mixing CR and RS in a ratio of 1:4 (v/v) was selected for the preparation of the colorimetric films.

**TABLE 1:** Color parameters (L*, a*, b* and △*E*) of anthocyanin solutions in different pH solutions.

pH	CR				RS				CR/RS1:1 (v/v)				CR/RS 1:4 (v/v)				CR/RS 4:1 (v/v)			
	L*	a*	b*	*ΔE*	L*	a*	b*	*ΔE*	L*	a*	b*	*ΔE*	L*	a*	b*	*ΔE*	L*	a*	b*	*ΔE*
2	79.44 ± 0.87	0.87 ± 0.13	24.57 ± 0.57	68.43 ± 0.14	72.74 ± 1.27	12.78 ± 0.22	5.53 ± 0.15	46.75 ± 0.05	74.87 ± 0.96	9.63 ± 0.24	15.33 ± 0.09	89.07 ± 0.16	68.74 ± 1.14	14.02 ± 0.15	13.13 ± 0.32	60.87 ± 0.11	76.43 ± 1.24	7.53 ± 0.07	18.69 ± 0.25	67.32 ± 0.08
4	79.89 ± 0.56	1.21 ± 0.22	25.68 ± 0.68	63.87 ± 0.17	75.17 ± 0.86	11.09 ± 0.27	4.0 ± 0.22	63.69 ± 0.07	74.19 ± 1.23	7.60 ± 0.22	23.13 ± 0.14	84.99 ± 0.22	70.35 ± 0.87	11.99 ± 0.21	14.26 ± 0.26	72.91 ± 0.15	78.15 ± 1.34	4.15 ± 0.06	21.26 ± 0.17	62.76 ± 0.04
6	80.07 ± 1.16	1.21 ± 0.28	23.61 ± 0.48	49.28 ± 0.07	78.71 ± 0.74	4.74 ± 0.37	1.32 ± 0.16	79.46 ± 0.12	75.76 ± 0.88	2.11 ± 0.11	25.52 ± 0.12	83.65 ± 0.08	75.79 ± 0.75	2.40 ± 0.08	14.23 ± 0.17	69.76 ± 0.08	78.62 ± 0.94	1.65 ± 0.06	21.06 ± 0.25	54.87 ± 0.11
7	79.37 ± 1.09	1.97 ± 0.18	21.33 ± 0.42	71.39 ± 0.09	74.09 ± 0.45	0.81 ± 0.17	−1.70 ± 0.11	72.03 ± 0.16	71.19 ± 0.68	1.30 ± 0.08	18.27 ± 0.23	71.23 ± 0.13	73.30 ± 0.58	−0.94 ± 0.02	5.60 ± 0.15	58.65 ± 0.05	76.75 ± 0.66	0.37 ± 0.02	13.28 ± 0.37	68.53 ± 0.05
8	68.35 ± 0.67	6.01 ± 0.37	3.27 ± 0.28	69.56 ± 0.11	72.73 ± 0.76	−1.13 ± 0.15	−0.62 ± 0.04	60.75 ± 0.34	67.66 ± 0.54	4.97 ± 0.12	5.16 ± 0.36	76.09 ± 0.08	67.71 ± 0.86	1.18 ± 0.06	0.63 ± 0.05	74.43 ± 0.10	71.42 ± 0.47	2.56 ± 0.04	0.95 ± 0.06	76.55 ± 0.12
10	66.93 ± 0.57	4.82 ± 0.28	0.74 ± 0.18	81.97 ± 0.15	71.25 ± 0.65	−1.25 ± 0.18	−2.29 ± 0.15	80.90 ± 0.26	68.69 ± 0.48	4.17 ± 0.21	4.63 ± 0.27	69.98 ± 0.05	68.61 ± 0.66	−2.26 ± 0.05	1.08 ± 0.03	83.76 ± 0.13	68.25 ± 0.67	2.11 ± 0.02	0.24 ± 0.04	65.39 ± 0.10
12	66.88 ± 0.46	8.69 ± 0.41	5.89 ± 0.28	67.54 ± 0.06	71.51 ± 0.91	−2.97 ± 0.25	−0.54 ± 0.06	68.69 ± 0.58	66.79 ± 0.81	6.99 ± 0.16	9.65 ± 0.36	55.43 ± 0.06	65.12 ± 0.54	0.40 ± 0.02	2.57 ± 0.06	65.44 ± 0.06	66.57 ± 0.78	5.56 ± 0.12	3.87 ± 0.03	74.23 ± 0.04
14	66.48 ± 0.58	9.13 ± 0.50	6.53 ± 0.33	56.90 ± 0.10	69.81 ± 0.59	−2.30 ± 0.31	−0.67 ± 0.07	56.48 ± 0.44	68.78 ± 0.77	6.66 ± 0.14	9.30 ± 0.33	72.87 ± 0.08	63.83 ± 0.39	1.26 ± 0.07	3.23 ± 0.08	76.90 ± 0.03	65.10 ± 1.05	7.13 ± 0.11	5.79 ± 0.15	53.76 ± 0.03

According to the results of Table 1, a decrease from +14.02 to +1.265 of the value of parameter a* indicated that the color of the mixing CR and RS in a ratio of 1:4 (v/v) solution changed from red to green, and a decrease from +13.134 to +3.233 of the value of parameter b* indicated that the color of the solution turned to blue. The parameters a* and b* were significantly different (*p* < .05) at pH 2–14; this further illustrated that the mixing CR and RS in a ratio of 1:4 (v/v) solution compared with the other four solutions was more sensitive to pH change and could be utilized as a pH‐sensitive mixed dye.

The visual color changes in the fabricated colorimetric film (mixing CR and RS in 1:4 (v/v) ratio) at various pH values are indicated in Figure 1f. The color was red at pH 2 and changed to green as the pH increased. A green color appeared at pH 14. Table 2 presented the color variables (L*, a*, b*, and △*E*) of the fabricated colorimetric film immersed in different pHs.

**TABLE 2:** Color parameters (L*, a*, b*, and △*E*) of the fabricated colorimetric film immersed in different pH.

pH	L*	a*	b*	*ΔE*
2	44.04 ± 1.57^d^	27.18 ± 0.26^a^	16.07 ± 0.45^b^	34.87 ± 0.65^a^
4	39.48 ± 1.37^b^	23.65 ± 0.55^b^	14.94 ± 0.61^d^	42.76 ± 0.47^e^
6	38.81 ± 0.67^a^	15.35 ± 0.49^c^	13.80 ± 0.52^f^	49.87 ± 0.28^f^
7	31.22 ± 0.83^c^	7.04 ± 0.08^d^	6.70 ± 0.11^b^	53.27 ± 0.64^d^
8	36.52 ± 0.47^e^	−0.77 ± 0.04^e^	−0.10 ± 0.04^a^	63.65 ± 0.72^c^
10	34.11 ± 0.68^f^	−1.33 ± 0.05^f^	1.08 ± 0.06^c^	58.34 ± 0.66^b^
12	31.42 ± 0.88^f^	5.69 ± 0.72^d^	1.44 ± 0.01^b^	48.94 ± 0.37^f^
14	16.34 ± 0.21^b^	6.06 ± 0.14^e^	−0.64 ± 0.03^c^	46.34 ± 0.57^d^

*Note*: Values are expressed as mean ± standard deviation. Different letters in the same column indicate significant differences (*p* < .05).

Chen et al. showed that the SPVA/glycerol film incorporated with curcumin and anthocyanins at a ratio of 2:8 (v/v) can be a good indicator material for the smart film for nondestructive monitoring of freshness of fish products (Xu et al., 2005).

### 3.2 FT‐IR spectra of films

**FIGURE 3:**
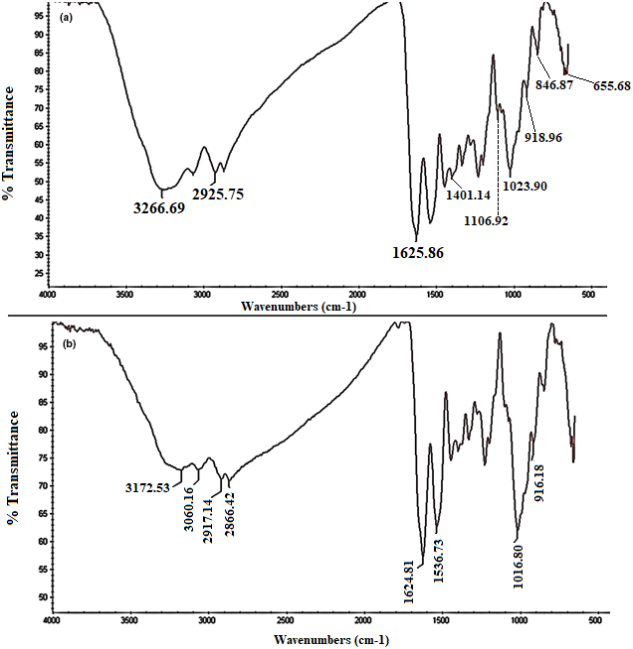
FT‐IR spectra of (a) pure gelatin film and (b) fabricated colorimetric film (mixing CR and RS in a ratio of 1:4 (v/v)).

Figure 3 shows FT‐IR spectra of the pure gelatin film and fabricated colorimetric film (mixing CR and RS in 1:4 (v/v) ratio). In the spectrum of gelatin film, the broadband at 3266 cm^−1^ and the peak at 1625 cm^−1^ were assigned to the stretching oscillation and bending oscillation of OH, respectively. The peak at 2925 cm^−1^ is related to the bending oscillation of CH. The characteristic peak appearing at 1626 cm^−1^ is characteristic of the tightly bound water present in gelatin. The bands from 655 to 1024 cm^−1^ correspond to the C‐O bond stretching (Pereira et al., 2015).

The spectrum for gelatin (Figure 3a) indicated the maximum absorption peak at 1106 cm^−1^ due to the C–O bond elongation and a peak at 1401 cm^−1^ corresponded to the bending vibrations of CH–CH_2_ which confirmed the basic carbon framework of gelatin (Zhai et al., 2017). In terms of fabricated colorimetric film spectrum (Figure 3b), the peaks between 2800 and 3300 cm^−1^, and the peaks at 1624 and 916 cm^−1^ are the three particular bands for carboxyl group identification, due to the stretching vibrations of O–H and C=O, and the out‐of‐plane bending vibrations of OH, respectively. The peak at 1536 cm^−1^ is associated with the combinations and overtones of aromatic compounds. Peak located at 1016 cm^−1^ indicates the presence of anthocyanin in the fabricated colorimetric film, which corresponds to aromatic ring C–H deformation (Wang, Cao, et al., 2019).

### 3.3 Physical properties of film

The physical characteristics of the film, such as water content, water solubility, swell index, thickness, and antioxidant properties of films are presented in Table 3.

**TABLE 3:** Moisture content, water solubility, swelling index, thickness, and antioxidant properties of the colorimetric films.

Film type	Moisture content (%)	Water solubility (%)	Swelling index (%)	Thickness (μm)	DPPH radical quenching (%)
G	20.56 ± 0.2^a^	45.67 ± 1.0^a^	132.45 ± 1.5^a^	44 ± 0.005^a^	–
G + CR‐RS (1:4 (v/v))	18.89 ± 0.32^h^	40.36 ± 1.1^g^	146.72 ± 1.3^c^	93 ± 0.008^d^	57.34 ± 1.3^i^

Abbreviations: G, Gelatin; DPPH, 2,2‐diphenyl‐1‐picrylhydrazy.

*Note*: Values are expressed as mean ± standard deviation. Different letters in the same column indicate significant differences (*p* < .05).

Most biopolymer‐based films are hydrophilic. Therefore, when foods are packaged with high water content, the film can absorb moisture and deteriorate, which limits its utilization in food packaging.

Overall, water solubilization and moisture amount are utilized as indicators to evaluate the moisture sensitivity of the active films. The results of water solubility and moisture amount of gelatin films with mixed natural dyes are shown in Table 1. The highest solubility in water and moisture content is related to the control sample, respectively. Increasing the natural dyes decreases this amount (*p* < .05).

In general, the purpose of biodegradable films is to create a protective outer layer for food packaging to increase the shelf life of the product and water resistance in food (Wei et al., 2017). It should also be noted that the addition of mixed natural dyes creates strong hydrogen bonds between the polymer matrix and the dyes. Because water molecules are not able to break the hydrogen bonds between dyes and the polymer matrix, therefore, the water solubility value of colorimetric film is less than the control sample (gelatin film).

Swelling index represents the water absorption capacity of the indicators during storage time, and significantly affects the color response performance of pH‐sensitive indicators because a higher swelling index leads to faster release (Moeinpour et al., 2019; Zhang, Liu, et al., 2019).

The results indicated that by the incorporation of mixed natural dyes, the indicator swelling index was increased. The abundant polyphenol molecules in RS and CR can decrease intermolecular interactions in gelatin and with hydrophilic groups of gelatins, such as amine groups and carboxyl groups forming hydrogen bonds, leading to a reduction in network bonding and an enhancement with swelling properties.

According to Table 3, the addition of natural dyes increases the thickness of the prepared film in comparison to the control sample (*p* < .05). In fact, the main reason leading to the increase in film thickness is owing to the high solids amount of the additive, which destroys the essential crystal structure of the film matrix, rising the spatial distance of the film matrix. Investigation of the antioxidant properties of packaging materials is also significant because it can slow down the chemical decomposition of packaged foods through oxidative reactions. Evaluation of antioxidant properties of gelatin film in the absence and presence of natural dyes by radical inhibitor method (DPPH). Gelatin packaging film showed no antioxidant properties, indicating that proteins and polysaccharides are not good radical scavengers. Adding a mixed solution of mixing CR and RS in a 1:4 (v/v) ratio to the film significantly enhanced their antioxidant activity. Research workers have reported that anthocyanins show powerful antioxidant activity due to the presence of multiple phenolic groups in their molecular structures (Karabagias et al., 2011).

### 3.4 Mechanical properties of films

Creating uniformity and integrity in composite films during processing is considered an important factor and protects food packaging as well as food items against incoming tension and stress (Nirmal & Benjakul, 2011). The tensile strength (TS), elongation at break (EB), and elastic modulus (EM) of the neat gelatin film were 40.07 ± 0.62 MPa, 20.25 ± 0.05%, and 53.15 ± 164.9 MPa, respectively, indicating that the gelatin film is relatively strong with fairly flexible and rigid. By the addition of CR‐RS (1:4 (v/v)), the strength (TS), and stiffness (EM) of the gelatin film decreased to 39.1 ± 0.23 MPa and 52.68 ± 0.32 MPa, respectively, but the decrease was not statistically significant (*p* > .05), while the flexibility (EB) increased significantly (*p* < .05) to 34.41 ± 0.19%. The tensile properties of blend films usually depend on the distribution and density of molecular interaction in the film matrix. The reduced strength and stiffness and increased flexibility of the gelatin/CR‐RS (1:4 (v/v)) film may be attributed to the plasticizing function of RS and CR, which can reduce the intramolecular forces between gelatin molecules and increase the mobility of the gelatin composite structure (Ezati & Rhim, 2020).

### 3.5 Morphology observation via FE‐SEM

**FIGURE 4:**
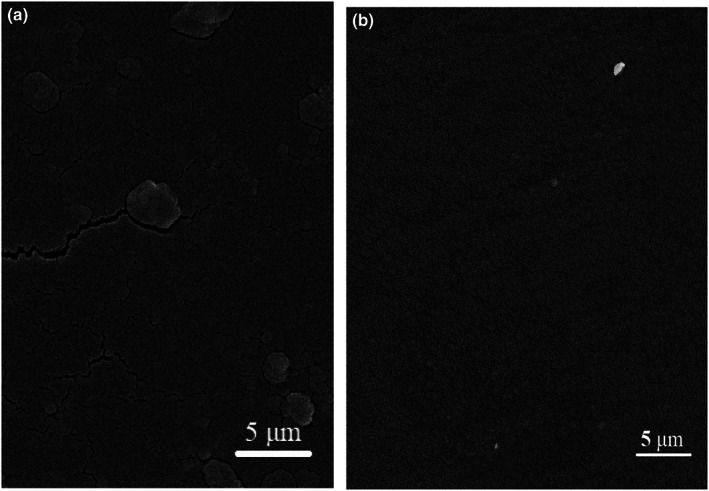
FE‐SEM images of the surface of (a) pure gelatin film and (b) fabricated colorimetric film (mixing CR and RS in a ratio of 1:4 (v/v)).

The surface morphology of gelatin and anthocyanin‐incorporated indicators are shown in Figure 4.

The surface films are shown in Figure 4. The pure gelatin film had a relatively smooth and uniform surface. However, it could be observed that there were cracks and folds dispersing in the gelatin film (Figure 4a). When CR‐RS (1:4 (v/v)) was incorporated into the gelatin film, the amount of small cracks and folds decreased. A few white spots presented on the surfaces of the gelatin/CR‐RS (1:4 (v/v)) film indicated some heterogeneity in the matrix when CR‐RS (1:4 (v/v)) was incorporated (Figure 4b) (Reddy & Rhim, 2014). In gelatin/CR‐RS (1:4 (v/v)) film, the surface was smooth and immaculate, implying that CR‐RS (1:4 (v/v)) had excellent compatibility with gelatin. Acting as plasticizers, anthocyanins contain several hydroxyl groups that can form intermolecular hydrogen bonds with the hydroxyl groups of the polymer matrix. Hydrogen bonds between CR‐RS (1:4 (v/v)) and the matrix contributed to the high compactness and cohesiveness developed film. Similar results have been reported by Koosha & Hamedi (2019).

### 3.6 Color response to volatile NH_3_

**FIGURE 5:**
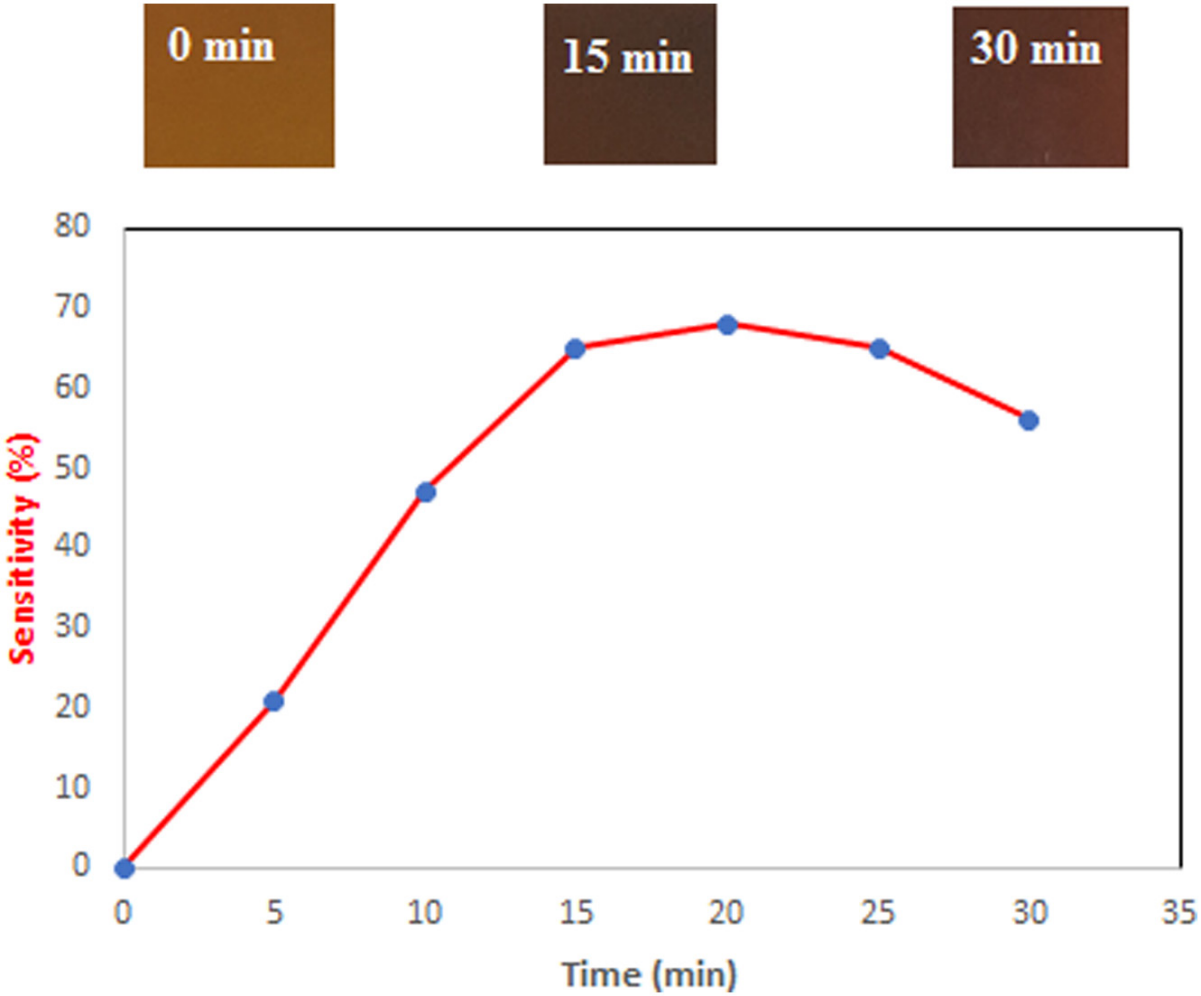
Ammonia sensitivity assay of the mixed natural dyes‐loaded film.

Colorimetric films react with the production of nitrogen‐based compounds (such as NH_3_) that can be utilized to check the freshness of high‐protein foods like meat and fish. Thus, the color of the mixed natural dyes‐loaded film was analyzed after exposure to NH_3_ for 30 min. The fastest color change was seen during the initial 15 min followed by a slower change for a longer time (Figure 5). The color of the film changes from reddish brown to dark brown and then brown. These color changes may be due to the development of a basic medium around the mixed natural dyes owing to the existence of the NH_3_ gas, which produces OH^−^ ions when it meets water.

### 3.7 Monitoring of shrimp spoilage

**FIGURE 6:**
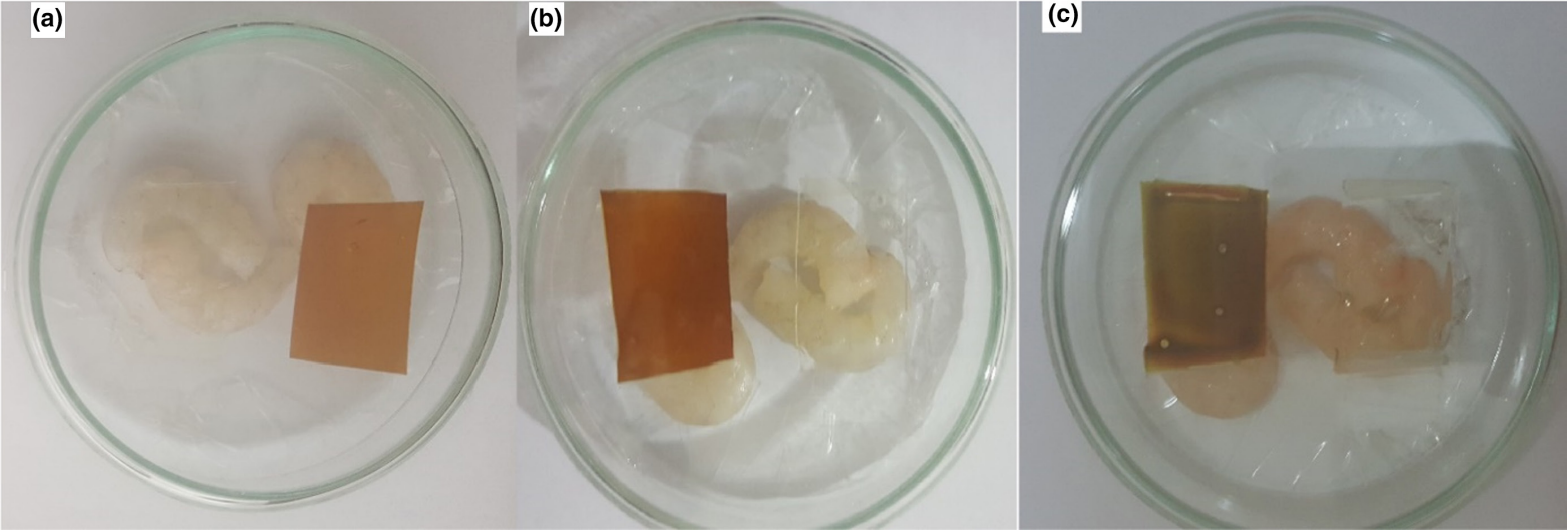
Color changes in colorimetric film attached to packages of shrimp, (a) immediately after packaging (b) 5 days and (c) 10 days of storage at 4°C.

As indicated on the package tag, all colorimetric films are placed inside the package without contacting the packaged shrimp samples, thereby an increase in volatile amine secretions from the shrimp. The various colors of colorimetric films immediately after packaging, 5 and 10 days of storage at 4°C could be seen in Figure 6. Film color changed when the shrimps were spoiled for 10 days and Δ*E* values in fresh shrimp, after 5 days and 10 days of storing at 4°C were 12.56, 16.85, and 41.27, respectively (*p* < .05). The designed colorimeter film offers two different colors related to freshness and shrimp spoilage that can be detected by the naked eye.

### 3.8 Changes in pH of the shrimp under storage

**FIGURE 7:**
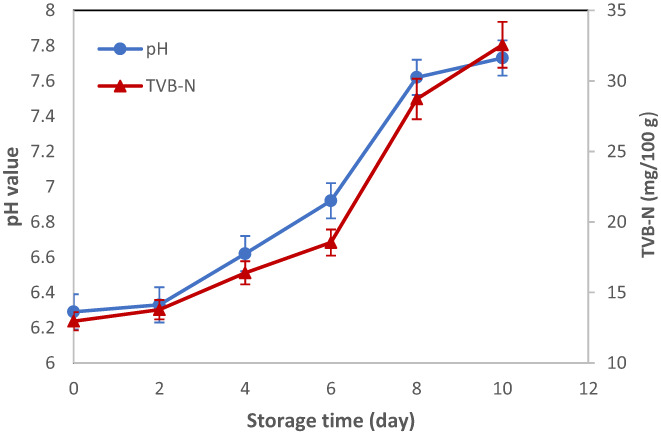
Changes in pH values and TVB‐N content of shrimp during refrigerated storage at 4°C.

pH changes can be used as a spoilage indicator in fishery products. The shrimp samples initially had a pH of 6.29 (day 0), but this value gradually increased during storage, reaching a value of pH 7.73 after 10 days of storage (Figure 7). Such results indicated the degree of meat spoilage through the decomposition of nitrogenous compounds, such as ammonia and trimethylamine mainly derived from microbial action, which affects the quality of the product during storage. Shrimps were not acceptable when the pH was greater than 7.6 (Koosha & Hamedi, 2019). This value exceeded the level for the control at day 8 of the storage. Kakaei et al. reported the pH values between 6.5 and 7.4 in minced trout fillets held at (4 ± 1°C) for 11 days (Kakaei & Shahbazi, 2016).

Mohebi et al. reported an increase in pH value from 6.23 to 7.91 for untreated shrimp after 11 days of storage in a refrigerator (4 ± 1°C) attributed to the protein breakdown by dominant microflora over time, which is in agreement with our findings (Mohebi & Shahbazi, 2017).

### 3.9 Total volatile base‐nitrogen (TVB‐N)

During the spoilage process, low‐molecular‐weight substances and alkaline compounds, particularly, ammonia and trimethylamine, which are generally known as TVB‐N, are produced and correspond to sensorial rejection and off‐flavors. TVB‐N is a distinctive index to assessshrimp spoilage. Samples with TVB‐N higher than 25 mg/l00 g are considered spoiled food (Nowzari et al., 2013). The initial TVB‐N value of shrimp was 12.96 mg/100 g, indicating the freshness of the samples. TVB‐N subsequently increased accompanied by pH during storage at 4°C (Figure 7). The increased trend of TVB‐N value can be attributed to protein degradation by microbial activity and autolytic enzymes resulting in the deamination of amino acids and accumulation of volatile bases (Huang et al., 2014). Spoilage of shrimp was verified with TVB‐N threshold of 28.72 mg/l00 g on day 8.

## 4 CONCLUSIONS

Five various potential smart indicator solutions were provided using natural dyes (RS, CR and mixture of RS and CR). Utilizing natural dyes solutions, in the pH range of 2–14, a change in color could be seen. The findings showed that the mixing CR and RS in 1:4 (v/v) ratio solution compared with the other four solutions indicated a larger color change with the naked eye and may be utilized as a pH‐sensitive mixed dye. The results of FE‐SEM and FT‐IR spectra showed that the mixed dyes were compatible with the film substrate. One of the salient features of the fabricated film is its color change at different pH, which can detect the process of spoilage of shrimp, and consumers can recognize the freshness of shrimp with the naked eye due to the change in color. The fabricated indicator film has advantages, such as easy preparation, ease of using, low cost, and usage of eco‐friendly substances.

## AUTHOR CONTRIBUTIONS

**Fatemeh S. Mohseni‐Shahri:** Conceptualization (equal); investigation (equal); methodology (equal); project administration (equal); supervision (equal); writing – original draft (equal); writing – review and editing (equal). **Farid Moeinpour:** Conceptualization (equal); investigation (equal); methodology (equal); project administration (equal); supervision (equal); writing – original draft (equal); writing – review and editing (equal).

## CONFLICT OF INTEREST STATEMENT

Fatemeh S. Mohseni‐Shahri declares that she has no conflict of interest. Farid Moeinpour declares that she has no conflict of interest.

## Data Availability

All data generated or analyzed during this study are included in this published article.
